# Mayaro Virus in Child with Acute Febrile Illness, Haiti, 2015

**DOI:** 10.3201/eid2211.161015

**Published:** 2016-11

**Authors:** John Lednicky, Valery Madsen Beau De Rochars, Maha Elbadry, Julia Loeb, Taina Telisma, Sonese Chavannes, Gina Anilis, Eleonora Cella, Massinno Ciccozzi, Bernard Okech, Marco Salemi, J. Glenn Morris

**Affiliations:** University of Florida, Gainesville, Florida, USA (J. Lednicky, V.M. Beau De Rochars, M. Elbadry, J. Loeb, E. Cella, B. Okech, M. Salemi, J.G. Morris, Jr.);; Christianville Foundation School Clinic, Gressier, Haiti (T. Telisma, S. Chavannes, G. Anilis);; Istituto Superiore di Sanita, Rome, Italy (E. Cella, M. Ciccozzi)

**Keywords:** Mayaro virus, arbovirus, viruses, Haiti, mosquito vector, vector-borne infections

## Abstract

Mayaro virus has been associated with small outbreaks in northern South America. We isolated this virus from a child with acute febrile illness in rural Haiti, confirming its role as a cause of mosquitoborne illness in the Caribbean region. The clinical presentation can mimic that of chikungunya, dengue, and Zika virus infections.

Mayaro virus (MAYV; genus *Alphavirus*, family *Togaviridae*) is a single-stranded positive RNA virus that was first isolated in Trinidad in 1954 (*1*) and is one of the viruses that comprise the Semliki Forest virus complex (*2*). Its transmission cycle is thought to occur mainly through mosquito vectors, especially those of genus *Haemagogus* (*3*), but *Aedes* spp. mosquitoes may also be competent vectors (*4*,*5*). The natural reservoirs of MAYV have been reported to be sylvatic vertebrates, mainly nonhuman primates but also birds and reptiles (*3*).

Since May 2014, when chikungunya virus (CHIKV) swept across the island of Hispaniola, researchers at the University of Florida (Gainesville, FL, USA) have studied alphavirus and flavivirus transmission in Haiti in collaboration with the Christianville Foundation. This foundation operates 4 schools in the Gressier/Leogane region of Haiti (≈20 miles west of Port-au-Prince) that serve ≈1,250 students from prekindergarten to grade 12 (*6*). The University of Florida has protocols in place for the collection of diagnostic blood samples from children seen at the school clinic with acute undifferentiated febrile illness (i.e., febrile illness with no localizing signs, such as would be expected with pneumonia, urinary tract infections, etc.).

From May 2014 through February 2015, blood samples were obtained from 177 children who met the criteria for acute undifferentiated febrile illness. The protocol for sample collection was approved by the University of Florida Institutional Review Board and the Haitian National Institutional Review Board. Written parental informed consent was obtained from parents or guardians of all study participants. Plasma samples were screened by reverse transcription PCR (RT-PCR) for CHIKV and dengue virus (DENV); samples that were negative for CHIKV were cultured by using cell lines and conditions as previously described (*7*). Zika virus and enterovirus D68 have been previously isolated from members of this school cohort (*7*,*8*). We report detection of MAYV in a child as part of this screening process.

## The Case

On January 8, 2015, an 8-year-old boy was examined at the school clinic because of fever and abdominal pain. His temperature was 100.4°F, lung sounds were clear, and his abdomen was soft and not tender. He had no rash and no conjunctivitis. On the basis of this clinical presentation, the clinic physician empirically diagnosed typhoid and administered co-trimoxazole.

A blood sample was collected, and RNA was extracted from plasma by using RT-PCR primers and the procedure described by Santiago et al. (*9*). The sample was negative for CHIKV but positive for DENV-1 (cycle threshold 26). In Vero E6 cells, which had been inoculated with the specimen, diffuse cytopathic effects typical for DENV-1 developed but at a much later time than for DENV-1–positive plasma specimens from other patients; this finding raised the possibility that DENV-1 had either mutated to reduced replication fitness or that the cells were co-infected with >2 incompatible viruses that were interfering with the replication of each other. DENV-1 viral RNA was detected by RT-PCR in the spent cell media of the plasma-inoculated cells but not in the spent media from noninoculated cells (negative control; Technical Appendix). Furthermore, no CHIKV- or Zika virus–specific amplicons were amplified from the spent media. However, apart from DENV-1, an alphavirus amplicon corresponding in size to that expected for MAYV was detected in viral RNA extracted from infected Vero cells. Sequencing confirmed that the amplicon corresponded to MAYV (GenBank accession no. KX496990).

The MAYV genome from Haiti was aligned with all MAYV strains available in GenBank, and a neighbor-joining tree was inferred from pairwise genetic distances estimated with the best fitting nucleotide substitution model (general time reversible plus gamma), as previously described (*7*). The phylogeny clearly shows 2 major and well-supported (bootstrap >90%) clades (Figure). The first clade includes strains sampled over the past 60 years from several South American countries (Peru, Bolivia, Venezuela, Trinidad and Tobago, and French Guiana); the second clade clusters the new Haiti strain with isolates from Brazil sampled during 1955–2014.

**Figure:**
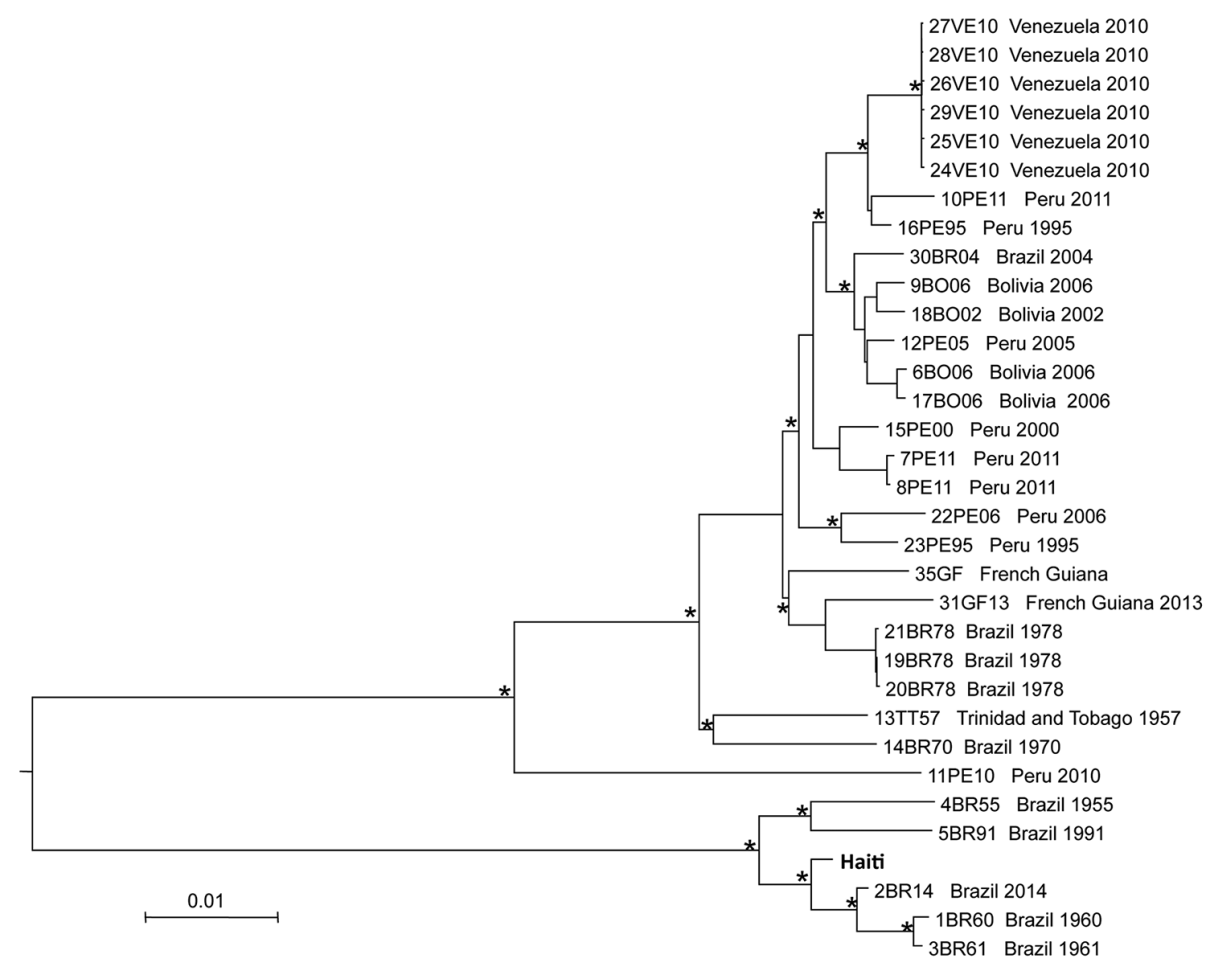
Neighbor-joining tree of full-genome Mayaro virus sequences. The tree was inferred from pairwise distances estimated with the best fitting nucleotide substitution model (general time reversible plus gamma). The tree includes the isolate from Haiti identified in this study (in boldface) and all full-genome sequences with known country of origin and sampling date downloaded from GenBank. Branches are drawn according to the scale bar at the bottom, which indicates nucleotide substitutions per site. An asterisk along a branch indicates bootstrap support >90% for the subtending clade.

## Conclusions

Although MAYV was originally isolated in Trinidad in 1954, subsequent reports of illness associated with this virus have tended to be associated with small, occasional outbreaks (30–100 cases) in northern South America (*10*,*11*), within and close to the Amazon forest. Signs and symptoms reported in association with MAYV infection include arthralgias, eye pain, fever, headache, myalgias, rash, and occasionally nausea and vomiting, photophobia, abdominal pain, cough, diarrhea, sore throat, and bleeding gums (*12*). A fatal infection associated with hemorrhagic fever has been reported (*13*). MAYV infections are probably underdiagnosed because of confusion with other mosquitoborne virus infections, especially dengue fever, which is endemic to the same areas. The emergence of CHIKV has further added to this confusion, especially because prolonged arthralgia is reportedly associated with CHIKV and MAYV infections (*3*).

Our findings suggest that MAYV is actively circulating in the Caribbean region and that there may be a link between the strain circulating in Haiti and the strains that have been circulating in Brazil since isolation of the virus in the 1950s. The patient from whom we isolated the organism had fever and abdominal pain but no rash or arthralgia. However, given that the patient was co-infected with DENV-1, it is difficult to separate out symptoms that are specific for MAYV infection. Of note, the clinic physician empirically diagnosed typhoid and treated the patient accordingly. The patient was from a rural/semi-rural area of Haiti, reflecting an ecologic setting that differs greatly from sylvan Amazon regions where many of the other reported MAYV infections have occurred. Little is known about vectors for MAYV in Haiti; potential animal reservoirs, if any, remain to be identified.

The recent emergence of Zika virus infection in the Caribbean region, and its identification as a major cause of birth defects, has brought a great deal of attention to arboviruses. Our findings highlight the multiplicity of arbovirus species in Haiti and the evolutionary relatedness among the viruses in Haiti and those circulating in Brazil, in keeping with prior work on Zika virus (*7*). Findings also underscore the complexity of the interactions among different species and the apparent proclivity for Zika virus/DENV (*7*) and MAYV/DENV co-infections. Although a better understanding of Zika virus infection is clearly needed, careful studies of other arboviruses (and their vectors and possible reservoirs) in these same geographic regions are correspondingly needed. We do not know if MAYV has epidemic potential; however, in light of recent observations with CHIKV, DENV, and Zika virus and the potential for transmission of MAYV by *Aedes* and *Haemagogus* spp. mosquitoes, inclusion of MAYV in studies of arbovirus transmission seems to be indicated.

Technical Appendix Additional methods used for virus isolation and sequencing.

## References

[1] Anderson CR, Downs WG, Wattley GH, Ahin NW, Reese AA. Mayaro virus: a new human disease agent. II. Isolation from blood of patients in Trinidad, B.W.I. Am J Trop Med Hyg. 1957;6:1012–6. 13487973 10.4269/ajtmh.1957.6.1012

[2] Powers AM, Brault AC, Shirako Y, Strauss EG, Kang W, Strauss JH, Evolutionary relationships and systematics of the alphaviruses. J Virol. 2001;75:10118–31. 10.1128/JVI.75.21.10118-10131.2001 11581380 PMC114586

[3] Llagonne-Barets M, Icard V, Leparc-Goffart I, Prat C, Perpoint T, André P, A case of Mayaro virus infection imported from French Guiana. J Clin Virol. 2016;77:66–8. 10.1016/j.jcv.2016.02.013 26921736

[4] Long KC, Ziegler SA, Thangamani S, Hausser NL, Kochel TJ, Higgs S, Experimental transmission of Mayaro virus by *Aedes aegypti.* Am J Trop Med Hyg. 2011;85:750–7. 10.4269/ajtmh.2011.11-0359 21976583 PMC3183788

[5] Smith GC, Francy DB. Laboratory studies of a Brazilian strain of *Aedes albopictus* as a potential vector of Mayaro and Oropouche viruses. J Am Mosq Control Assoc. 1991;7:89–93. 1646286

[6] Beau De Rochars VE, Alam MT, Telisma T, Masse R, Chavannes S, Anilis MG, Spectrum of outpatient illness in a school-based cohort in Haiti, with a focus on diarrheal pathogens. Am J Trop Med Hyg. 2015;92:752–7. 10.4269/ajtmh.14-0059 25732684 PMC4385768

[7] Lednicky J, Beau De Rochars VM, El Badry M, Loeb J, Telisma T, Chavannes S, Zika virus outbreak in Haiti in 2014: molecular and clinical data. PLoS Negl Trop Dis. 2016;10:e0004687. 10.1371/journal.pntd.0004687 27111294 PMC4844159

[8] El Badry M, Lednicky J, Cella E, Telisma T, Chavannes S, Loeb J, Isolation of an enterovirus D68 from blood of a child with pneumonia in rural Haiti: close phylogenetic linkage with New York strain. Pediatr Infect Dis J. 2016 Jun 21. Epub ahead of print. 10.1097/INF.0000000000001283 27331858

[9] Santiago GA, Vergne E, Quiles Y, Cosme J, Vazquez J, Medina JF, Analytical and clinical performance of the CDC real time RT-PCR assay for detection and typing of dengue virus. PLoS Negl Trop Dis. 2013;7:e2311.. 10.1371/journal.pntd.0002311 PMC3708876 23875046

[10] Muñoz M, Navarro JC. Mayaro: a re-emerging arbovirus in Venezuela and Latin America [in Spanish]. Biomedica. 2012;32:286–302. 10.1590/S0120-41572012000300017 23242303

[11] Suhrbier A, Jaffar-Bandjee M-C, Gasque P. Arthritogenic alphaviruses—an overview. Nat Rev Rheumatol. 2012;8:420–9. 10.1038/nrrheum.2012.64 22565316

[12] Tesh RB, Watts DM, Russell KL, Damodaran C, Calampa C, Cabezas C, Mayaro virus disease: an emerging mosquito-borne zoonosis in tropical South America. Clin Infect Dis. 1999;28:67–73. 10.1086/515070 10028074

[13] Navarrete-Espinosa J, Gómez-Dantés H. [Arbovirus causing hemorrhagic fever at IMSS] [in Spanish]. Rev Med Inst Mex Seguro Soc. 2006;44:347–53. 16904038

