# Correction: Vol. 22, No. 11

**DOI:** 10.3201/eid2304.C22304

**Published:** 2017-04

**Keywords:** errata, erratum, correction

The name of author Massimo Ciccozzi was misspelled in Mayaro Virus in Child with Acute Febrile Illness, Haiti, 2015 (J. Lednicky et al.). The article has been corrected online (https://wwwnc.cdc.gov/eid/article/22/11/16-1015_article).

