# Supplementary material for: Mayaro Virus in Child with Acute Febrile Illness, Haiti, 2015
Source: Emerg Infect Dis. 2016 Nov;22(11):2000–2. doi: 10.3201/eid2211.161015 (PMC5088037; doi:10.3201/eid2211.161015)
Supplement: Technical Appendix — Additional methods used for virus isolation and sequencing. [file 16-1015-Techapp-s1.pdf]

Article DOI: http://dx.doi.org/10.3201/eid2211.161015

# Mayaro Virus in Child with Acute Febrile Illness, Haiti, 2015

## Supplemental Material

## Viral Isolation/Sequencing

Virus DNA (vDNA) and RNA (vRNA) were extracted from spent cell growth media and, separately, from the virus-infected cells. Spent cell growth media was centrifuged at low speed to pellet cellular debris, and the clarified spent media treated with cyanase nuclease to degrade nucleic acids external to that packaged (and thus protected) in virions using a Nucleic Acid Removal Kit (RiboSolutions, Inc., Cedar Creek, Texas), and vDNA and vRNA co-extracted from the treated material using a QIAamp Viral RNA Mini Kit (Qiagen, Valencia, CA, USA) (Lednicky et al). In contrast, for the virus-infected cells, total RNA was extracted by an RNeasy kit (Qiagen), following the manufacturer's instructions.

*PCR of vDNA purified from spent cell media:* PCR for DNA viruses was performed using primers specific for the detection of Epstein Barr virus and cytomegalovirus, and polyomaviruses (information provided upon request). PCR was performed with One Taq DNA polymerase (New England Biolabs, Ipswich, MA, USA)

*RT-PCR of vRNA purified from spent cell media:* Real-time RT-PCR (rtRT-PCR) was performed for CHIKV, DENV, and ZIKV using the primers systems discussed in Lednicky et al (*1*). Standard RT-PCR was performed using virus group-specific primers that included universal α- and flavivirus primers (*2*) and universal primers for other RNA viruses (arena-, bunya-, corona-, paramyxo-, picorna-, and rubella viruses, information provided upon request). Briefly, a High-Capacity cDNA Reverse Transcription Kit (Applied Biosytems, ThermoFisher Scientific, Wyman, MA, USA) primed with non-ribosomal hexamers (0.6 mM) in the presence of SUPERase-In RNase inhibitor (Ambion, Austin, TX) was used for cDNA synthesis. Noteworthy, as an additional precaution, non-ribosomal hexamers were used was used to favor the reverse transcription of viral genomes over rRNA (*3*), even though the vRNA had already been pre-

treated using the RiboSolutions kit components, which is designed to remove contaminating rRNA. PCR was performed with One Taq DNA polymerase (New England Biolabs). Amplicons from standard RT-PCR reactions were analyzed on a 2% agarose gel stained with ethidium bromide (EtBr).

*RT-PCR of RNA purified from virus-infected cells:* A High-Capacity cDNA Reverse Transcription Kit (Applied Biosytems) was used with non-ribosomal hexamers (as described above) in the presence of SUPERase-In RNase inhibitor, and rtRT-PCR or RT-PCR performed using the CHIKV, DENV, and ZIKV described in Lednicky et al [1], the α- and flavivirus primer systems of de Morais et al (*2*), and RT-PCR systems for arena-, bunya-, corona-, paramyxo-, picorna-, and rubella viruses, as mentioned above. No MAYV strains/isolates of nucleic acid were present in the laboratory at the of the study; MAYV genomic sequences have never been detected in any work in our laboratory until the present study, and the cell lines and mock infected cells do not have Mayaro virus sequences.

*Sequencing of MAYV:* For RT-PCR, first-strand synthesis was performed using non-ribosomal hexamers and Accuscript High Fidelity 1st strand cDNA kit (Agilent Technologies, Santa Clara, CA, USA in the presence of SUPERase-In RNase inhibitor. PCR was performed with Phusion Polymerase (New England Biolabs) with denaturation steps performed at 98°C. A gene walking approach with overlapping primers was used (Technical Appendix Table), with most of the specific amplicons in the 800 bp range. A 5′ and 3′ system for the Rapid Amplification of cDNA Ends (RACE) was used per the manufacturer's protocols (Life Technologies, Carlsbad, CA, USA) to obtain the 5′ and 3′ ends of the MAYV genome. The reverse primer used for 5′ reaction consisted of MAYV-5R-RACE-JAL (5′-CCTGCTGTGCTTCCACTTCAAATGC-3′), and the 3′ forward primer was: MAYV-3F-RACE-JAL (5′-GGTCACCTATCCGGCAAATCACAACG-3′). Additionally, for 3′ RACE, 5′-$T_{25}$A-3′ was used as the reverse primer. Virus-specific PCR amplicons were purified, sequenced bidirectionally using Sanger Sequencing, and the sequences assembled with the aid of Sequencher DNA sequence analysis software v2.1 (Gene Codes, Ann Arbor, MI, USA). The GenBank accession number is KX496990.

## References


1. Lednicky J, Beau De Rochars VM, El Badry M, Loeb J, Telisma T, Chavannes S, et al. Zika virus outbreak in Haiti in 2014: molecular and clinical data. PLoS Negl Trop Dis. 2016;10:e0004687. PubMed http://dx.doi.org/10.1371/journal.pntd.0004687

2. de Morais Bronzoni RV, Baleotti FG, Ribeiro Nogueira RM, Nunes M, Moraes Figueiredo LT. Duplex reverse transcription-PCR followed by nested PCR assays for detection and identification of Brazilian alphaviruses and flaviviruses. J Clin Microbiol. 2005;43:696–702. PubMed http://dx.doi.org/10.1128/JCM.43.2.696-702.2005

3. Endoh D, Mizutani T, Kirisawa R, Maki Y, Saito H, Kon Y, et al. Species-independent detection of RNA virus by representational difference analysis using non-ribosomal hexanucleotides for reverse transcription. Nucleic Acids Res. 2005;33:e65. PubMed http://dx.doi.org/10.1093/nar/gni064


**Technical Appendix Table.** Primers used for sequencing of MAYV Haiti/1236/2015*

| Primer | Primer nucleotide sequence, 5′→3′† | Nucleotide position‡ |
|---|---|---|
| 5R-RACE-JAL | CCTGCTGTGCTTCCACTTCAAATGC | 182–158 |
| MAYV-1F | CGCCGGACGTCTCTAAGCTCTTCCT | 36–61 |
| MAYV-1R | CACTGACGGTAAATGCCAACTC | 877–856 |
| MAYV-2F | GCAACCAATTGGGCTGACGAACAG | 674–697 |
| MAYV-2R | GACTGCAGAAGAAGCCTAATTCGG | 1509–1486 |
| MAYV-3F | GGTCCGCAGGGTTGTCGATAGGAATCAG | 1455–1482 |
| MAYV-3R | CACGCCCAAGACCCCGACCAC | 2257–2237 |
| MAYV-4F | CGAAGACCGGCGGCCCCGTAC | 2207–2227 |
| MAYV-4R | GGTTGCTGTAAAATTCCCTTTTGG | 3034–3011 |
| MAYV-5F | GTGATCCCTGGATCAAGACCTTGAG | 2979–3003 |
| MAYV-5R | GGTAGTGGTGTAGCCTATACGGTGTG | 3779–3754 |
| MAYV-6F | CGCTGGCAGGTACGACCTGGTCTTC | 3718–3742 |
| MAYV-6R | CGCGGCAGTAGATAGTGACCCGTGC | 4523–4499 |
| MAYV-7F | CAATCACTCTCGCATCTGTTGGC | 4457–4459 |
| MAYV-7R | GGGCGAGGCGGCGGTATGGGA | 5271–5251 |
| MAYV-8F | CCTACGCCAACGGCAGAACTTGC | 5222–5244 |
| MAYV-8R | CGTCTACCATATCCAAGTATGCGTC | 6041–6017 |
| MAYV-9F | GGCGTCCTACCAGATTACGGATG | 5989–6011 |
| MAYV-9R | CGATAAGGTCCAATAGCTGGTTATC | 6794–6770 |
| MAYV-10F | GTTGATGATATTGGAGGACCTG | 6742–6763 |
| MAYV-10R | GGGCCTGTTGGTCTGGTCTCTGCATTG | 7555–7529 |
| MAYV-11F | CCACCACGCCCCTTGGAGACCAC | 7498–7519 |
| MAYV-11R | GAATTTACGTTCTCCTCCAGCATCCTCAG | 8351–8323 |
| MAYV-12F | GCTGTGCACCGTGCTGCTATGAAAAGG | 8282–8308 |
| MAYV-12R | GCCATTTTGTGTGGCTCGTGACGTAAGCCTG | 9121–9091 |
| MAYV-13F | CCATTAATAGCTGTACCGTTGAC | 9062–9084 |
| MAYV-13R | GTGCTCGTAAGCACTGGCAACG | 9870–9849 |
| MAYV-14F | GTCGCAATGAGCATCGGGAGTG | 9826–9847 |
| MAYV-14R | CGCCTTGGCGTTAAGCGGTGAGTC | 10626–10603 |
| MAYV-15F | CCCTATACCCAGACTCCATCTGGC | 10555–10578 |
| MAYV-15R | GTTATTATATGCGCCATTAGAGACG | 11374–11350 |
| 3F-RACE-JAL | GGTCACCTATCCGGCAAATCACAACG | 11016–11041 |
| $T_{25}A$ | TTTTTTTTTTTTTTTTTTTTTTTTTA | polyA tail |

*MAYV, Mayaro virus.
†Mismatches with corresponding nucleotide in MAYV BR/SJRP/LPV01/2015 (GenBank accession no. KT818520.1) indicated in red.
‡Nucleotide position in MAYV BR/SJRP/LPV01/2015 and in MAYV Haiti/1236/2015.
